# The Cancer Epitope Database and Analysis Resource (CEDAR)

**DOI:** 10.1093/nar/gkac902

**Published:** 2022-10-17

**Authors:** Zeynep Koşaloğlu-Yalçın, Nina Blazeska, Randi Vita, Hannah Carter, Morten Nielsen, Stephen Schoenberger, Alessandro Sette, Bjoern Peters

**Affiliations:** Center for Infectious Disease and Vaccine Research, La Jolla Institute for Immunology, La Jolla, CA, USA; Center for Infectious Disease and Vaccine Research, La Jolla Institute for Immunology, La Jolla, CA, USA; Center for Infectious Disease and Vaccine Research, La Jolla Institute for Immunology, La Jolla, CA, USA; Department of Medicine, University of California San Diego, La Jolla, CA, USA; Department of Bio and Health Informatics, Technical University of Denmark, Lyngby, Denmark; Instituto de Investigaciones Biotecnológicas, Universidad Nacional de San Martín, San Martín, Argentina; Laboratory of Cellular Immunology, La Jolla Institute for Immunology, La Jolla, CA, USA; Center for Infectious Disease and Vaccine Research, La Jolla Institute for Immunology, La Jolla, CA, USA; Department of Medicine, University of California San Diego, La Jolla, CA, USA; Center for Infectious Disease and Vaccine Research, La Jolla Institute for Immunology, La Jolla, CA, USA; Department of Medicine, University of California San Diego, La Jolla, CA, USA

## Abstract

We established The Cancer Epitope Database and Analysis Resource (CEDAR) to catalog all epitope data in the context of cancer. The specific molecular targets of adaptive T cell and B cell immune responses are referred to as epitopes. Epitopes derived from cancer antigens are of high relevance as they are recognized by anti-cancer immune cells. Detailed knowledge of the molecular characteristic of cancer epitopes and associated metadata is relevant to understanding and planning prophylactic and therapeutic applications and accurately characterizing naturally occurring immune responses and cancer immunopathology. CEDAR provides a freely accessible, comprehensive collection of cancer epitope and receptor data curated from the literature and serves as a companion site to the Immune Epitope Database (IEDB), which is focused on infectious, autoimmune, and allergic diseases. CEDAR is freely accessible at https://cedar.iedb.org/.

## INTRODUCTION

Adaptive immune responses recognizing cancer cells can play an essential role in clinical outcomes of disease, contributing to partial or complete inhibition of tumor growth. These responses can be naturally occurring or induced/amplified by deliberate therapeutic immunization (1,2). Prophylactic vaccines against hepatitis B virus (HBV) and human papillomavirus (HPV) have successfully been applied to prevent liver and cervical cancer, respectively (3,4). Conversely, adaptive immune responses can also contribute to immunopathology, as illustrated by the relatively common incidence of Immune-Related Adverse Events (irAEs) associated with Immune Checkpoints Blockade (ICB) therapy (5). It is therefore of paramount interest to be able to define and study cancer-specific and cancer-related adaptive immune responses at the molecular level.

Adaptive immunity relies on two main types of responses; one is mediated by B cells, while the other is mediated by T cells. In the context of cancer responses, B cells engage cancer antigens expressed on the surface of cancer cells. Furthermore, in certain cases, B cells also respond to secreted antigens or cytoplasmic antigens derived from destroyed cancer cells by secreting antibodies that are capable of interfering with cancer growth or killing cancer cells by various mechanisms (6–10). T cells recognize peptides derived from proteins that are degraded intracellularly and loaded onto a Major Histocompatibility Complex (MHC) on the cell surface (11,12). Two primary types of T cells are involved in cancer-specific responses; CD8 and CD4 T cells. CD8 ‘killer’ T cells recognize epitopes in the context of class I MHC molecules. They can recognize and kill cancer cells and secrete various inflammatory cytokines causing bystander damage to tumor cells. CD4 ‘helper’ T cells recognize epitopes in the context of class II MHC molecules. They orchestrate cancer-specific antibody responses, secrete various inflammatory cytokines, and are also capable of direct cytotoxicity (13,14).

Receptors on the surface of B cells (BCRs) and T cells (TCRs) bind to specific parts of these antigens, called epitopes, which is a necessary step for B cell and T cell activation and proliferation. Cancer antigens include products of mutated genes, overexpressed proteins, cancer germline antigens, cell type-specific differentiation antigens, antigens produced by oncogenic viruses, and altered glycolipids and glycoproteins (15). Each antigen category has generated interest in developing antigen-targeting immunotherapies, which only yielded limited success in past clinical trials (reviewed in (1). It is known that tumor-specific T cells are often dysfunctional due to inhibitory signals at the tumor site (15). Immune-checkpoint blockade (ICB) therapies, such as inhibiting CTLA-4 or PD-1, can remove these inhibitory signals and reinvigorate T cells (16). With the advent of ICB therapies, interest in antigen-based immunotherapies has resurrected. When combined with ICB, antigen-based vaccines, for example, can elicit strong and durable immune responses (2). Neoantigens, i.e. antigens encoded by somatic mutations, are of specific interest as they are highly tumor-specific and not expressed on normal cells.

The resurgence of interest emphasizes the need to catalog all cancer epitope-related data linked to the biological, immunological and clinical contexts and, most importantly, make this information freely available to the scientific community in a user-friendly format. There is also a need to develop resources for computational epitope prediction and analysis tools that provide researchers access to predictive strategies and objective evaluations of their performance. There have been several recent efforts to address these needs. The TANTIGEN 2.0 database (17) contains curated epitope and ligand elution data for many different cancer antigens, such as neoantigens and differentiation antigens, but does not include peptides that were shown to be ineffective and also lacks any association with clinical data. Similarly, the Cancer Antigenic Peptide Database (https://caped.icp.ucl.ac.be) also only includes curated epitope data for several different cancer antigens. NEPdb (18) contains curated neoantigens but lacks any other types of cancer antigens. For cataloged neoepitopes, associated receptor information and clinical data are also provided if available. It is possible to query NEPdb for an epitope sequence of interest, but there is no option to search for receptors. dbPepNeo (19) only contains curated HLA class I restricted neoantigens and ligand elution data. Notably, while all resources provide some essential tools to query the databases for cancer types and peptide sequences, it is not possible to perform specific and granular queries. the Immune Epitope Database (IEDB) has been in existence since 2003 and comprehensively catalogs epitope data for allergy, infectious disease, transplantation, and autoimmunity but does not include cancer (20).

We have established the Cancer Epitope Database and Analysis Resource (CEDAR) as a companion site to the IEDB. Analogous to the IEDB, CEDAR includes all cancer-specific epitope data from various T cell and B cell experiments, MHC binding assays, and MHC ligandomics by mass-spectrometry. CEDAR provides a freely accessible, comprehensive collection of cancer epitope and receptor data curated from the literature and is accessible under https://cedar.iedb.org/.

### Curation process

**Figure 1.**
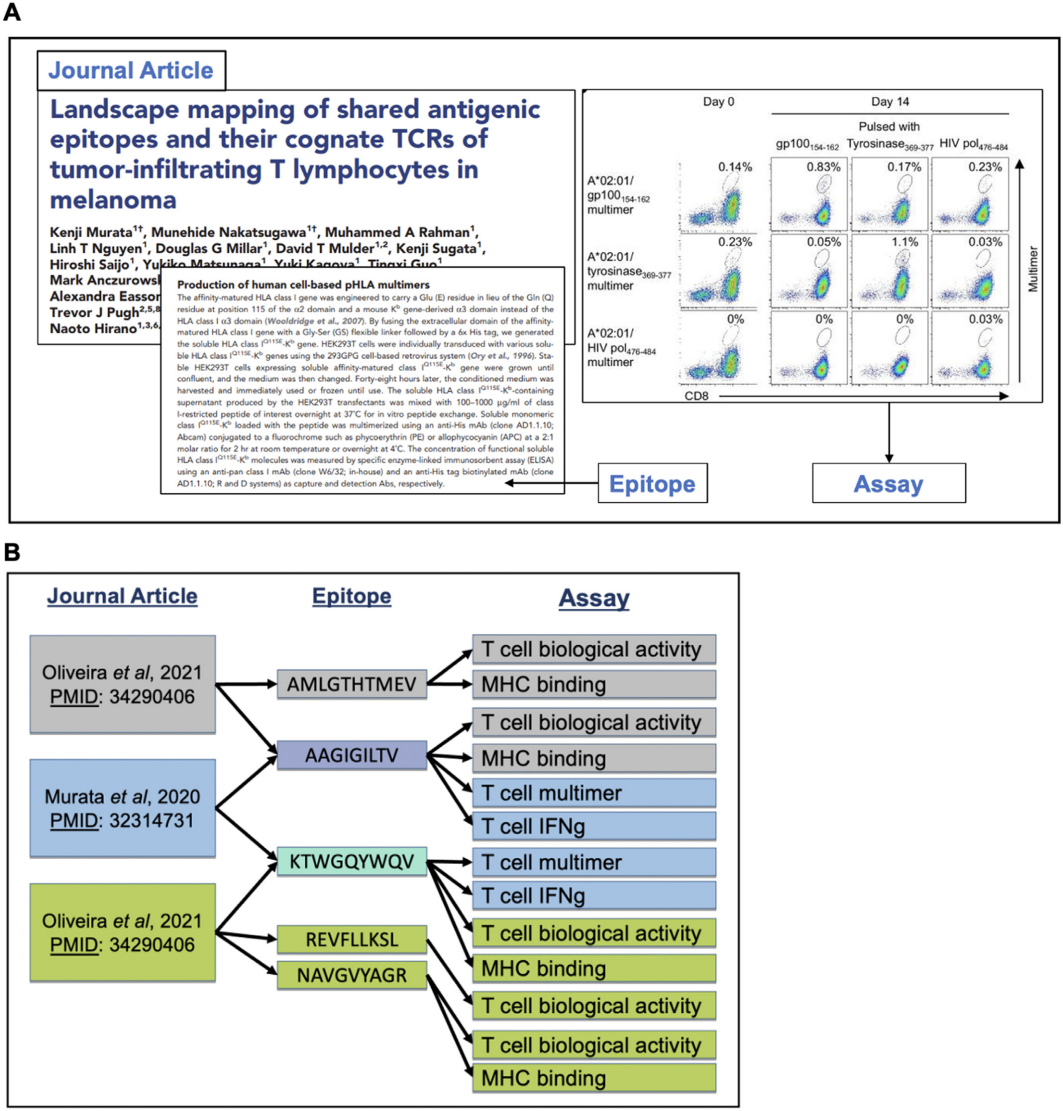
(**A**) Curation is performed on a per publication basis. All epitope-specific data and details of the assay used to describe the immune response are captured (33). (**B**) Any curated reference in CEDAR may describe multiple epitopes that may have been tested in multiple different assays. In turn, any single epitope captured in CEDAR may have been described in multiple references and in various contexts.

CEDAR is populated by manual curation of the cancer literature, following detailed curation guidelines, and is assisted by automated validation (21). Following a workflow developed and extensively validated in the case of the IEDB over the course of 19 years and analysis of >23 000 records, the curation process begins with an automated PubMed query that retrieves potentially relevant publications. The retrieved abstracts are categorized as being one of several cancer categories by a document classifier, as previously described (22). Journal articles are read by a PhD-level curator who identifies all experimental assays where an adaptive immune receptor was tested for recognition of an epitope, including both negative and positive outcomes. All assay types that provide epitope-specific recognition data are captured, including those giving antibody and TCR sequences and generating 3D structures. All epitope-specific data and details of the assays used to describe the immune responses are captured (Figure 1A). Data is entered into a web form that ensures consistency by incorporating external ontologies, inter-field validation, and auto-complete functionalities (21,23). Data accuracy is maintained via adherence to the curation guidelines mentioned above and detailed extensively in a publicly accessible curation manual (http://curationwiki.iedb.org/wiki/index.php/Curation_Manual2.0). Additionally, we employ a peer review process whereby a second curator will read the publication and review the data entered by the first curator. They will work together to ensure the data is accurately represented before making the record public. Once approved, curated records are made public by our weekly build process (21).

Curation is performed on a per publication basis, with all data, including positive and negative outcomes, from each publication curated fully as a stand-alone record. Thus, any single epitope may have been described in multiple papers and in various contexts (Figure 1B); for example, one paper may study the antibody response to a given epitope in the context of immunization with purified protein of a healthy mouse, while another paper may study the antibody response to the same epitope, spontaneously occurring in the context of in a human cancer patient. As part of this curation process, epitopes are assigned a source antigen—if they are directly derived from a naturally occurring protein—and an accompanying source organism. Cancer-associated antigens are identified as those that include epitopes recognized specifically in hosts that have cancer.

### Identification of antigens and antigen subtypes in CEDAR

**Figure 2.**
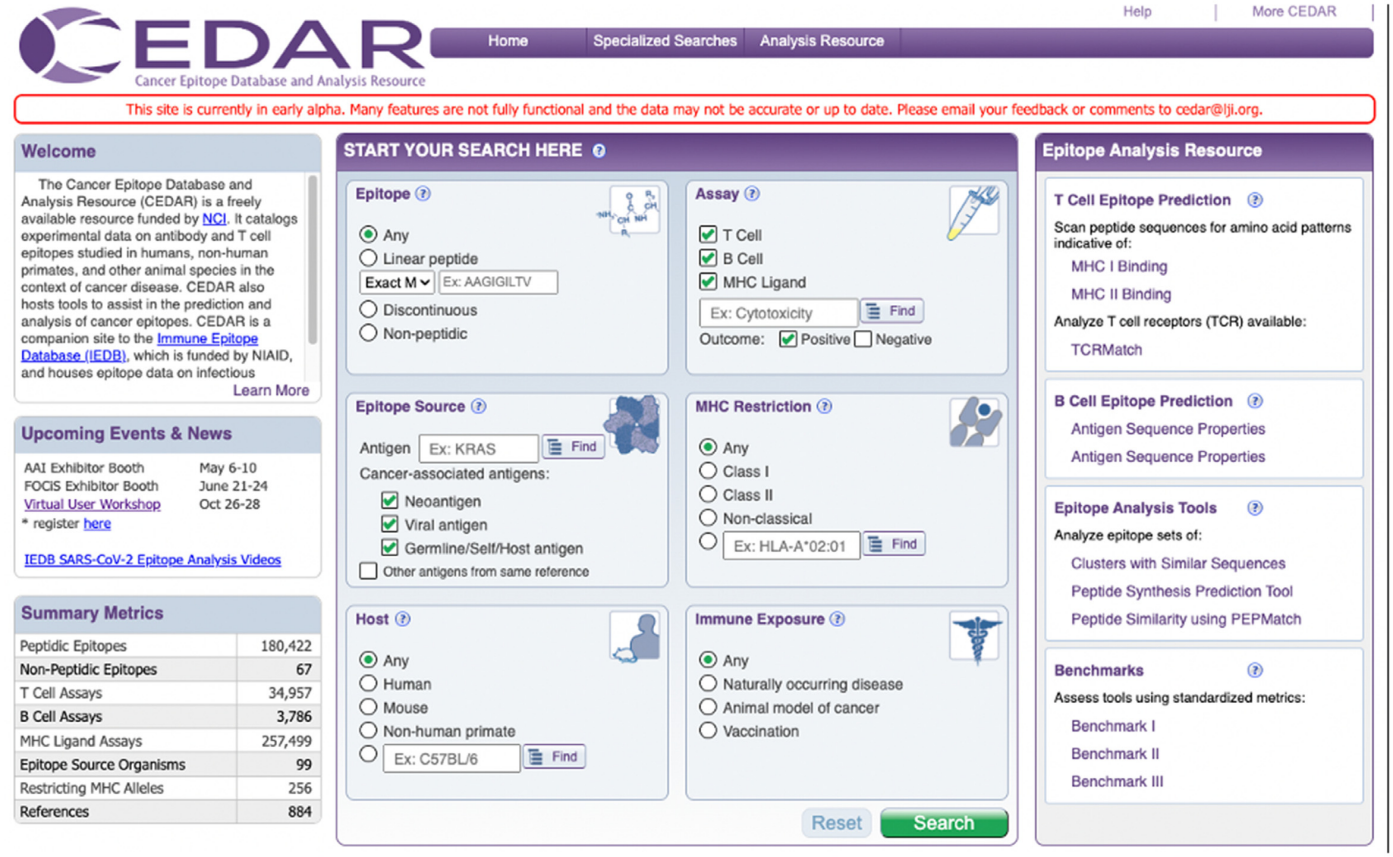
CEDAR search interface.

All cancer-associated antigens are considered when curating epitopes for CEDAR, including mutated antigens (e.g. neoantigens), oncoviral antigens (e.g. HPV), cancer germline antigens (e.g. MAGE, PRAME, NY-ESO-1), differentiation or tissue-specific antigens (e.g. MART-1, gp100, CEA, prostate antigens), and overexpressed antigens (e.g. Her2/neu, Survivin, wildtype p53) (15). However, these categories are not mutually exclusive, as there are multiple ways in which the same set of tumor antigens may be classified (24). We defined three broader categories of cancer-associated antigens that can be clearly distinguished and are mutually exclusive, namely ‘Neoantigen’, ‘Viral antigen’ and ‘Self-antigen’ (Figure 2). We also include antigens that are not cancer-associated, if they were reported together with cancer-associated antigens in a cancer-related study (‘Other antigens from same reference). These antigens are clearly flagged and can be excluded when querying CEDAR (Figure 2).

We developed the following approach to classify each epitope into one of these four categories. From all the cancer-related references in CEDAR, we first establish a list of cancer-associated antigens, which consists of all antigens that were reported in a cancer-associated assay and that generated a positive response. Antigens present in cancer-related papers but that are not in the cancer-related antigen list are grouped into the category ‘Other antigens from same study’. Epitopes for which the ‘related object type’ was curated as being ‘neo-epitope’, are grouped into the category ‘Neoantigen’. Every epitope, that is not a neoantigen, is derived from a virus, and is derived from a cancer-associated antigen is classified as ‘Viral antigen.’ If the antigen source organism is a vertebrate and is derived from a cancer-associated antigen, it is considered a ‘Germline/Self/Host antigen’.

### Search Interface

One of the challenges for biomedical databases is to develop query interfaces that are intuitive and still allow the user to perform granular queries. To ensure this, we conducted interviews with several experts in the cancer immunology research field. During several iterations, we developed a search interface that focuses on making the most requested pieces of information immediately accessible (Figure 2). We repurposed several fields that are also used in the IEDB search interface for epitope structure, host, assays used to characterize the response, and MHC restriction. These fields are well established and proven to be essential for many epitope queries. We also introduced cancer-specific fields in CEDAR, such as the option to select specific cancer-associated antigen subtypes as the epitope source, including ‘Neoantigen’, ‘Viral antigen’ and ‘Germline/Self/Host antigen’. Antigens that are not cancer-associated but were reported together with cancer-associated antigens in a cancer-related study can also be included in the query by selecting ‘Other antigens from same reference. The default selection on the CEDAR homepage is to exclude those antigens and only query all cancer-associated antigens.

Users can also search for epitopes encoded by specific genes (e.g. Prostate Specific Antigen). Another set of fields allows the user to search for epitopes in the context of a certain immune response induction, such as ‘Naturally occurring disease’, ‘Animal model of cancer’ or ‘Vaccination’. In the example in Figure 2, we searched for MHC class I restricted T cell epitopes derived from prostate-specific antigen in humans with naturally occurring disease. Executing this search queries the entire CEDAR content, i.e. all epitope, antigen, assay, and receptor records, and returns records meeting the current search criteria.

### Results display

**Figure 3.**
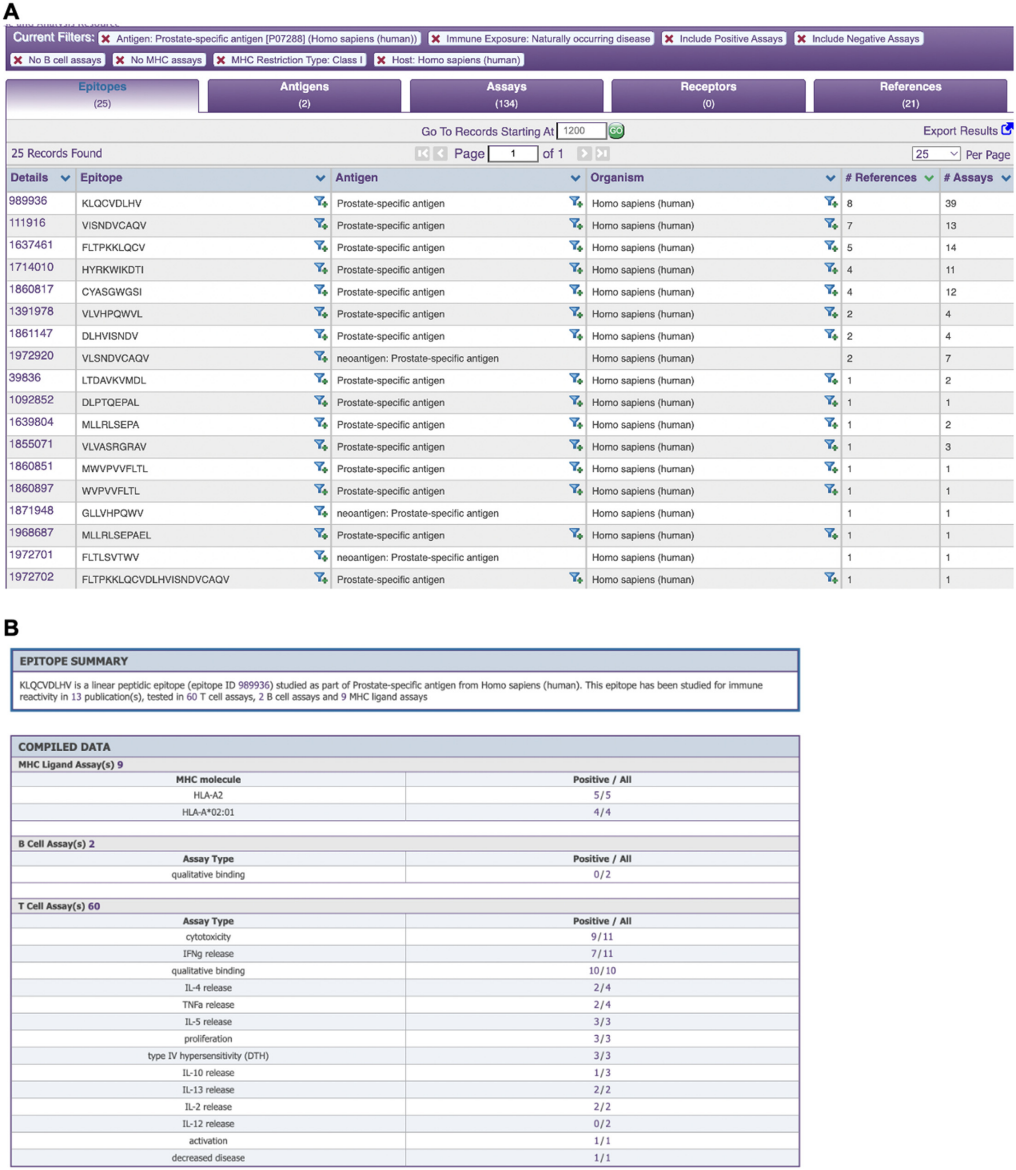
CEDAR results presentation of epitope data. (**A**) The ‘Epitope’ tab displays one unique epitope per row together with its source antigen and organism. For each epitope, all available assays and references are summarized as numbers. (**B**) ‘Epitope Details’ page provides information on all experimental contexts an epitope was tested in with a textual summary of the compiled data and in data tables. For each assay type the epitope was tested in, it displays how often it was tested, the outcome (i.e. positive or negative), and links to these assays.

Once a query has been executed, the search results are presented on a new page (Figure 3A). The results are grouped in four tabs: Epitopes, Antigens, Assays, Receptors, and References that match the current search criteria. The search criteria are displayed at the top of the results table and any filter can also be removed at this stage. Additional search panels added to the left side of the page allow the current results to be further refined by adding search parameters.

The ‘Epitopes’ tab, for example, displays one unique epitope per row together with its source antigen and organism. For each epitope, all available assays and references are summarized as numbers. If the user is interested in a specific epitope from this list, results can be filtered by clicking on the siphon symbol next to it. This will filter all records in the tabs Epitopes, Antigens, Assays, Receptors, and References to only show records related to the epitope of interest.

By clicking on the Epitope ID in the ‘Details’ column of an epitope a new browser tab containing the ‘Epitope Details’ page is opened (Figure 3B). The Epitope Details page provides information on all experimental contexts an epitope was tested in with a textual summary of the compiled data at the top of the page and in the data tables below. Each assay type, i.e. MHC ligand, B cell and T cell assay, is presented in a separate section of the data table and provides a summary of assay subtypes the epitope was tested in, how often it was tested, the outcome (i.e. positive or negative), and links to these assays. Using the Epitope Details page, a user can quickly form opinions regarding an epitope of interest. For example, the assays performed on the epitope in Figure 3B suggest that the epitope KLQCVDLHV binds to HLA-A*02:01 and can activate T cells as shown by several cytotoxicity, cytokine release, and proliferation assays.

**Figure 4.**
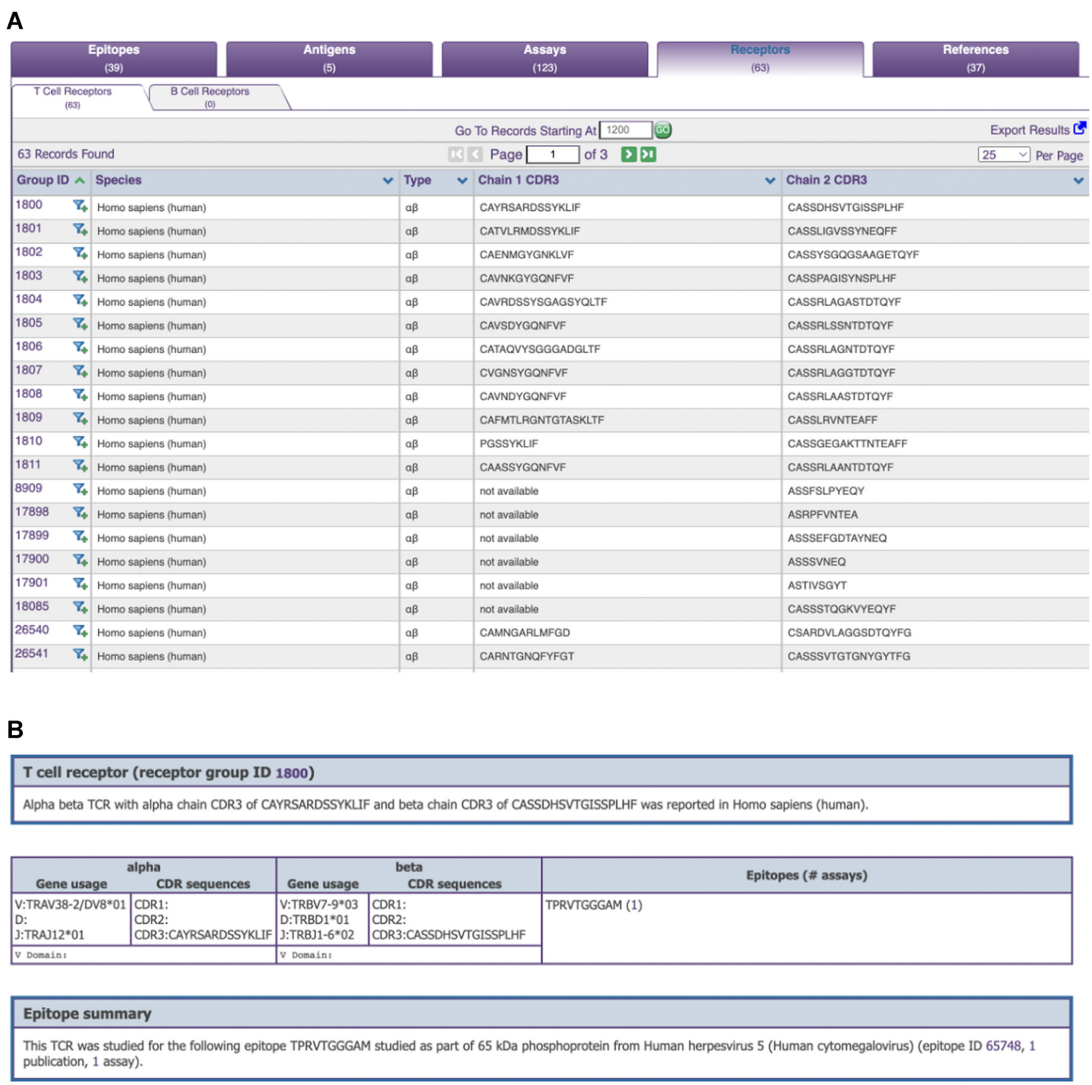
CEDAR results presentation of receptor data. (**A**) The ‘Receptor’ tab has two sub-tabs for T cell and B cell receptors. In each row of the TCR or BCR tab, a unique receptor is displayed along with the species it was reported in, the type, and the Chain 1 and Chain 2 CDR3 sequences, if available. (**B**) The ‘Receptor Details’ page provides relevant information about the species that the receptor was reported in and links to the 3D structures in PDB. In the data table below, the gene usage and sequences for CDR1, CDR2 and CDR3, as well as the full-length receptor sequence are displayed if available for both alpha and beta chains. Sequences of the epitopes that the receptor was reported to recognize are also listed, together with links to the corresponding epitopes in CEDAR.

The ‘Receptor’ tab has two sub-tabs for T cell and B cell receptors that meet the current search criteria (Figure 4A). In each row of the TCR or BCR tab, a unique receptor is displayed along with the species it was reported in, the type (e.g. αβ TCRs), and the Chain 1 and Chain 2 CDR3 sequences, if available. Analogous to the ‘Epitope’ tab, details about a receptor can be retrieved by clicking on its ID in the ‘Group ID’ column. On the ‘Receptor Details’ page, relevant information about the species that the receptor was reported in is displayed, and links to the 3D structures in PDB are summarized at the top of the page (Figure 4B). In the data table below, the gene usage and sequences for CDR1, CDR2 and CDR3, as well as the full-length receptor sequence are displayed if available for both alpha and beta chains. Sequences of the epitopes that the receptor was reported to recognize are also listed, together with links to the corresponding epitopes in CEDAR.

### Downloading data from CEDAR

After executing a query, the corresponding results from each of the tabs, Epitopes, Antigens, Assays, Receptors and References, can be downloaded by clicking on ‘Export Results’ in the upper right corner of the results display. The user has the option to include Internationalized Resource Identifiers (IRIs) or not. IRIs are identifiers that resolve uniquely and unambiguously to records both within and outside of CEDAR.

All data stored in CEDAR can also be retrieved as a bulk download. The download page can be accessed from the homepage by clicking on ‘More CEDAR’ on the top right corner, and then selecting ‘Database Export’ from the pop-up. Complete database exports are available in XML and MySQL formats. Most users prefer to download data in a tabular format, which can be found under the section ‘CSV Metric Exports’. Here, the user has the option to separately download T cell (tcell_full_v3.zip), B cell (bcell_full_v3.zip) and MHC ligand elution assays (mhc_ligand_full.zip). These tables contain all information on the assay level and an epitope will be listed multiple times if it was reported in the context of multiple different assays.

### Public databases utilized by CEDAR to ensure accuracy and interoperability

To accurately represent epitope information, CEDAR utilizes community-supported ontologies for most of its specific fields. NCBITaxonomy (25) is used to describe the host and epitope source organisms, UniProt (26) to describe proteins and ChEBI (27) for non-peptidic structures. The Ontology for Biomedical Investigations (OBI) (28) describes assay types, and the MHC Restriction Ontology (MRO) (29) is used for MHC alleles, Cell Ontology (30) for annotating responder, stimulator, and effector cell types, Uberon (31) for tissue types and the Disease Ontology (32) for describing disease states.

### Metrics on data included in CEDAR

In the planning stages of the CEDAR resource, PubMed literature searches were conducted to broadly categorize possibly curatable cancer references. The references identified and their corresponding experimentally-derived epitopes can be broadly classified into the following areas (15); mutated (e.g. neoantigens), oncoviral (e.g. HPV), cancer germline antigens (e.g. MAGE, PRAME, NY-ESO-1), differentiation or tissue-specific antigens (e.g. MART-1, gp100, CEA, prostate antigens), and overexpressed antigens (e.g. Her2/neu, Survivin, wildtype p53). The curation of cancer-related data for CEDAR commenced in 2021 with the neoepitope category. This category was selected due to the importance and renewed interest in neoepitopes for cancer therapies, such as checkpoint blockade and immunotherapy. As of 31 July, we have curated over 16 100 neoepitopes from 200 references. Currently, 94 references are outstanding for curation in this category as we have added new references identified in literature searches, by our collaborators and the broader user community.

In parallel, we chose to curate epitopes from prostate-associated differentiation and tissue-specific antigens. We chose this antigen category because it encompasses a limited number of well-studied and well-characterized antigens such as prostatic acid phosphatase (PAP), prostate specific antigen (PSA) and prostate specific membrane antigen (PSM), and the data can be used to visualize the CEDAR capabilities as it relates to a large data volume associated with specific antigens. As of 31 July, we have curated over 540 prostate-associated epitopes from 159 references. Currently, eight references are outstanding for curation in this category, reflective of a steady state where new references appearing in the literature are continuously introduced in the curation pipeline. Next, we plan to start curating epitopes from selected cancer germline antigens such as the melanoma-associated antigen gene family (MAGE). As mentioned above, with the success of checkpoint blockade therapies, there is renewed interest in these antigens as potential targets for immunotherapy.

### Conclusion and future plans

We have established The Cancer Epitope Database and Analysis Resource (CEDAR). CEDAR provides a freely accessible, comprehensive collection of cancer epitope and receptor data curated from the literature. Overall, as of 31 July 2022, we have curated a total of 224 355 cancer-associated epitopes from 875 references. These epitopes have been tested in almost 317 000 assays (34 889 T cell assays, 3780 B cell assays and 278 068 MHC ligand elution assays). Further to this, over 3600 receptors have been curated; 3526 T cell receptors and 108 antibodies. We currently have ∼3100 possibly curatable cancer references still outstanding in our curation pipeline.

While CEDAR’s first alpha site is fully functional, we have several plans to improve CEDAR’s search interface. We envision including the option to search for neoantigens encoded by a specific mutation. We also anticipate adding a characterization of the neoplasm/tumor, which will allow the user to search for epitopes reported in a specific cancer type. Furthermore, we plan to provide the ability to select specific methods that were used to induce immune responses. Currently, it is not possible to search for receptor sequences directly from the main CEDAR search interface, but it is only possible to filter for specific sequences once a query has been executed. We plan to provide this option directly on the main CEDAR search interface in the future. Finally, we also plan to provide an Application Programming Interface (API) to allow users to perform custom and more complex queries.

### Data Availability Statement

All data is deposited in cedar.iedb.org.

## Notes

Present address: Center for Infectious Disease and Vaccine Research, La Jolla Institute for Immunology, 9420 Athena Circle, La Jolla, CA, USA.
